# The Lethal Combination of Pregabalin With Other Recreational Drugs

**DOI:** 10.7759/cureus.81675

**Published:** 2025-04-03

**Authors:** Alison Muñoz, Marai Roque, Marco Valladares, Kim Nguyen, Eric Tran, Guinda St. Fleur, Lorraine Mukona, Nadia Yar, Mohammed Siddiqui

**Affiliations:** 1 Family Medicine, Chino Valley Medical Center, Chino, USA; 2 Family Medicine, Chino Valley Medical Center, chino, USA; 3 College of Medicine, Chino Valley Medical Center, Chino, USA

**Keywords:** delusions, drug abuse, ketamine, lyrica, recreational drugs, toxic encephalopathy

## Abstract

This case presents a 33-year-old male with a four-day history of worsening altered mental status and rhabdomyolysis. He experienced delusions, restlessness, and visual hallucinations, exacerbated by prolonged multidrug abuse, including over-the-counter pregabalin (Lyrica) for pain relief and recreational ketamine. Despite an initially incomplete history and unclear etiology, the patient was admitted for toxic-metabolic encephalopathy with acute kidney injury (AKI) and required significant medical management for confusion, acidosis, and electrolyte disturbances. This patient’s severe presentation serves as a caution against self-medicating with neuromodulators like pregabalin, especially alongside current or historical misuse of other neuroactive substances. This case affirms the necessity of swiftly discerning both clinical symptoms and psychiatric risk factors in the effective identification and treatment of dangerous pregabalin intoxication.

## Introduction

In the last decade, the use of the prescription drug pregabalin has steadily risen in favor as a treatment for epilepsy and various forms of neuropathic pain [1]. Its off-label uses include anxiety, restless legs syndrome, chronic pruritus, and menopausal vasomotor symptoms [2]. Additionally, pregabalin is a stabilizer and is used in the treatment of various psychiatric diseases. It is also commonly used in managing different kinds of addictions. In tandem with pregabalin’s rising popularity, there is also increasing concern regarding its misuse for sensations of euphoria, altered consciousness, or self-medicated pain relief, particularly among those with a history of drug abuse. Pregabalin belongs to a class of drugs called gabapentinoids, whose mechanism of action involves the inhibition of certain neurotransmitters throughout the central nervous system [1]. Although generally well tolerated, pregabalin use at high doses or in combination with other drugs can lead to serious complications such as delirium, encephalopathy, rhabdomyolysis, respiratory distress, and hallucinations [2]. Some of the adverse effects during therapeutic use include dizziness, drowsiness, visual disturbances, and weight gain. Oftentimes, drug users seek pregabalin for its euphoric effects or to reduce withdrawal symptoms. For these reasons, accurate toxicological monitoring is required for these high-risk individuals.

## Case presentation

A 33-year-old Caucasian male with a history of depression and a prior suicide attempt by drug overdose was brought to the emergency room on July 9, 2024, due to four days of worsening altered mental status. Because the patient was disoriented at the time of admission, history was provided by his parents, and arterial blood gas (ABG) was obtained (Table 1), revealing that he had been experiencing delusions, restlessness, and visual hallucinations.

**Table 1:** Arterial Blood Gas

Type of Lab for ABG	Normal ABG Lab Values	Lab Values
pH	7.35-7.45	7.23
pCO_2_ at Pt Temp	35-45 mmHg	32.4
pO_2_ at Pt Temp	75-100 mmHg	51.4 (P)
HCO_3_	22-26 mEq/L	13.3
O_2_ Sat (measured)	95-100%	82.6
Base excess	Varies	-13.0
Carboxyhemoglobin	<2%	0.3 (N)
Methemoglobin	<2%	0.4 (N)

Over a week prior, he traveled from his home in Costa Rica to the United States for the first of bilateral arthroscopic hip surgeries due to injuries from long-distance running. His prescription medications included Zoloft 100 mg daily (QD) and Wellbutrin (unknown dose QD). In January 2024, his bilateral hip injuries impeded his ability to run, and the patient reported increased depression due to the loss of this exercise. He began taking self-prescribed Lyrica (pregabalin) 200 mg, available over the counter in Costa Rica, for pain relief and admitted that he equated it to Tylenol, taking up to eight pills daily. The patient’s history is significant for past prescription and illicit drug abuse, along with a two-month history of recreational ketamine use every other day. He denied alcohol use. At the time of admission, physical examination was significant for tachycardia, with a pulse of 105 beats per minute and minimal response to verbal stimuli.

In the Emergency Department (ED), the patient underwent a significant workup, including labs and imaging mentioned below. His initial labs revealed elevated liver function tests (LFTs), ammonia, and severely elevated creatine kinase. The patient had an unremarkable head computed tomography (CT), and an electrocardiogram (EKG) showed a right bundle branch block. Despite concerns for drug abuse and intoxication, his urine drug screen and blood alcohol levels were both negative.

The patient was admitted for toxic encephalopathy with acute kidney injury (AKI) and rhabdomyolysis. Throughout his admission, he was given lactulose 30 mL three times daily (TID) for hyperammonemia, intravenous (IV) thiamine 500 mg every eight hours (Q8), and IV fluids with isotonic bicarbonate for uremic acidosis and hyperkalemia (Table 2). Treatment was continued despite confirmation of whether the cause of hyperammonemia was due to hepatic dysfunction, renal failure, or muscle breakdown.

**Table 2:** Lab Values for Entire Hospital Admission CK: creatine kinase, AST: aspartate aminotransferase, ALT: alanine aminotransferase, BUN: blood urea nitrogen.

Lab Ordered	Normal Value	Lab Value on 07/09	Lab Value on 07/10	Lab Value on 07/11	Lab Value on 07/12	Lab Value on 07/13	Lab Value on 07/14	Lab Value on 07/15	Lab value on 07/16	Lab Value on 07/17
CK	55-170 IU/L	2634	5373	8440	4472	1503	3270	3371	1352	618
Ammonia	15-45 mg/dL	179	119	88	69	90	34	19	11	20
AST	8-48 IU/L	110	116	160	130	70	92	97	66	None taken
ALT	10-50 IU/L	70	64	77	78	66	67	69	69	None taken
Creatinine	0.7-1.3 mg/dL	1.6	1.5	1.1	1.1	0.9	0.7	0.6	0.7	None taken
BUN	6-20 mg/dL	37	31, 24	19	12	12	8	7	10	None taken
K^+^	3.6-5.2 mmol/L	5.4	4.9, 3.7	3.3	3.7	3.3	3.0	2.9	3.5	None taken
Mg_2_^+^	1.7-2.2 mg/dL	2.5	None taken	2.3	1.8	1.9	1.7	1.7	2.2	None taken
Ca_2_^+^	8.5-10.2 mg/dL	9.2	8.0	8.6	8.7	8.4	8.9	8.8	8.4	None taken

Despite days of interventions, the patient continued to have an altered mental status and increasing agitation. A neurologist was consulted and clonazepam was ordered as needed (pro re nata). A brain magnetic resonance imaging (MRI) was negative for acute stroke, and lumbar puncture yielded insignificant, colorless cerebrospinal fluid (CSF) (Table 3).

**Table 3:** Lumbar Puncture Fluid Analysis

CSF Fluid Property	CSF Fluid Normal Values and Description	CSF Fluid Patient Values and Description
Pressure	5-20	6.0
Appearance	Clear/colorless	Clear
Color	Colorless	Colorless
WBC (white blood cell)	<5 cells	3 cells
RBC (red blood cell)	< 1	163
Glucose (mg/dL)	50-80	62
Total protein (mg/dL)	15-45	41.9

Although the patient appeared to slowly improve in both mental and motor function, he remained confused, with slow speech and disorientation to situations. On July 14, he attempted to leave the hospital on three occasions despite reorientation by family and nursing staff. Throughout his admission, the patient’s creatine kinase fluctuated, and by July 15, a nephrologist recommended discontinuing haloperidol in case it had contributed to the elevation. Within 24 hours, the patient’s creatine kinase (CK) decreased and continued trending down, along with his levels of ammonia, aspartate aminotransferase (AST), alanine aminotransferase (ALT), and creatinine.

The patient returned to full baseline mental status on July 17, and a psychiatrist psychologically cleared him for discharge. Upon discharge, the patient was counseled at length regarding drug misuse and overdose and expressed understanding. He was instructed to follow up with his primary care physician (PCP) within one week and to continue outpatient psychiatry follow-up. No medications were prescribed at discharge.

## Discussion

Pregabalin is a γ-aminobutyric acid (GABA)-derived medication approved in the United States for the treatment of adult patients with epilepsy or pain related to diabetic neuropathy, fibromyalgia, or postherpetic neuralgia [1]. It binds to voltage-gated calcium channels present throughout the central nervous system (CNS), inhibiting the release of excitatory neurotransmitters like glutamate and norepinephrine [1,2].

Numerous studies in the last decade show that gabapentinoids like pregabalin have increasing abuse potential due to their sedating, euphoric effects and ease of accessibility online and on the black market [2]. Additionally, reports of pregabalin abuse are markedly increased in patients with current or previous dependence on other substances, especially users of opiates and sedatives who use it to potentiate their effects. The higher bioavailability of pregabalin also contributes to its increased potency over gabapentin [3]. These rising concerns are multinational, as seen in a Northern Ireland-based study of pregabalin fatalities, stating that these “deaths [in NI] are generally seen in young men, especially 30-39-year-olds, many of whom have a history of substance misuse” [4,5]. Though therapeutic dosing of pregabalin can vary significantly based on its intended use, the general maximum dose considered safe is around 300-600 mg/day. For example, when prescribed for neuropathic pain, the general pregabalin dosing recommendations are 25 mg daily (QD) or 50-150 mg/day distributed in two to three doses. Conversely, the patient followed in this case was self-medicating for pain with pregabalin doses of up to 1600 mg/day.

Before the patient presented in the ED, his family’s initial concerns for his health stemmed from noticing increased fatigue and decreased appetite for a few days.

The general side effects of pregabalin to keep in consideration are noted as CNS depression (e.g., somnolence and dizziness), increased risk of suicidal ideation, peripheral edema, visual disturbances, and weight gain [4]. Serious respiratory depression can become life-threatening, most often with accompanying use of other CNS depressant medications, such as opioids, benzodiazepines, or antidepressants [5]. Some studies have shown that multiple drug users and those in methadone treatment programs often administer pregabalin at high dosages to achieve euphoria, reduce withdrawal symptoms, or potentiate the effects of methadone [6]. Recent reports have also noted that pregabalin abuse has become particularly prevalent among individuals seeking an alternative to opioids or other hard-to-obtain substances, raising concerns in clinical practice, particularly with black market sales of pregabalin [7].

Symptoms of rhabdomyolysis result from the release of intracellular elements from necrotic muscle into the circulation and are typically characterized as a triad of myalgias, muscle weakness, and reddish-brown urine. Yet, patients can be asymptomatic, and this full triad may be observed in only 1-10% of cases [5]. This patient’s rhabdomyolysis presentation of lethargy, dark urine, and a severely elevated creatine kinase level with metabolic acidosis did not initially raise concern for pregabalin-specific toxicity, and diagnoses such as hepatorenal failure were still being considered. However, pregabalin-induced rhabdomyolysis has been reported in rare cases and is thought to be somewhat associated with the kidneys’ role in pregabalin clearance from the body [5,8]. In a similar fashion to this case, other studies have found pregabalin cessation and a combination of hydration and diuresis treatments to resolve patients’ rhabdomyolysis [1].

Caution must be exercised when considering the use of pregabalin in patients at increased risk of respiratory distress (e.g., those with lung disease or advanced age) [5], renal impairment, or a history of psychiatric conditions or substance abuse. Although gabapentinoids in general have also been specifically prescribed as a beneficial option for pain relief in those with opioid dependence [1].

Prescribers must also be aware of the pharmacokinetic properties that make pregabalin more likely to be dangerously abused than other common gabapentinoids like gabapentin. Pregabalin is absorbed in the body much more quickly, reaching peak blood concentrations within an hour, compared to gabapentin’s approximate three-hour onset [1]. Unless safety concerns require more rapid removal, pregabalin is usually withdrawn over the course of a week to minimize withdrawal symptoms, which may include delirium, agitation, seizures, and more. However, excessively disrupting the normal balance and utilization of these neurotransmitters can directly result in drug-induced delirium and worse. Adverse side effects of pregabalin include visual disturbances, weight gain, respiratory distress, and CNS depression [2]. While patients on appropriately prescribed doses may experience dizziness and drowsiness, pregabalin intoxication combined with other drugs can quickly progress to dangerous encephalopathy, hallucination, rhabdomyolysis, and other potentially fatal complications.

Post-marketing surveillance data suggest that pregabalin abuse is particularly common among individuals with prior substance use disorders, with polydrug use exacerbating the problem. Pregabalin is often combined with sedatives, opioids, and alcohol, which amplifies its addictive effects and heightens the risk of severe toxicity [8]. Pregabalin abuse has been associated not only with physical dependence but also with behavioral addiction, especially in patients with a history of opioid addiction or other psychoactive substance use. As such, it is crucial for clinicians to monitor its use in patients with such backgrounds carefully. Prescribers must take thoughtful precautions in using pregabalin in patients with a history of angioedema, renal impairment, lung disease, and substance abuse [9].

## Conclusions

The complexity of altered mental status presents well-known diagnostic challenges, as emphasized in this case’s difficult determination of which of the many CNS-affective substances in the patient’s possession were contributing most to his intoxication. It is imperative that physicians, whether initiating the outpatient treatment of chronic pain or managing detoxification or withdrawal in an inpatient setting, are cognizant of the rising abuse potential and risk of serious complications with pregabalin. The diverse list of physicians consulted on the cause of this patient’s encephalopathy illustrates the importance of multidisciplinary communication and partnership in acute patient care. This could potentially open up more research and hospital resources to initiate pregabalin testing in urine samples. It can also raise the question of the necessity of inpatient detoxification or early psychiatry involvement during the hospital course. Overall, physicians should become more familiar with pregabalin and understand how to safely prescribe it and educate patients.
